# Potassium *N*-bromo-2,4-dichloro­benzene­sulfonamidate sesquihydrate

**DOI:** 10.1107/S1600536812042456

**Published:** 2012-10-20

**Authors:** B. Thimme Gowda, Sabine Foro, H. S. Spandana

**Affiliations:** a Department of Chemistry, Mangalore University, Mangalagangotri 574 199, Mangalore, India; b Institute of Materials Science, Darmstadt University of Technology, Petersenstrasse 23, D-64287 Darmstadt, Germany

## Abstract

The asymmetric unit of the title salt, K^+^·C_6_H_3_BrCl_2_NO_2_S^−^·1.5H_2_O, contains one K^+^ cation, one *N*-bromo-2,4-dichlorobenzenesulfonamidate anion, one water molecule in general position and one water molecule located on a twofold rotation axis. The K^+^ cation is hepta-coordinated by three water O atoms and four sulfonyl O atoms from three symmetry-related *N*-bromo-2,4-dichloro­benzene­sulfonamide anions. The S=N distance of 1.575 (3) Å is consistent with that of a double bond. In the crystal, the anions are linked by O—H⋯Br and O—H⋯N hydrogen bonds into layers parallel to the *ac* plane.

## Related literature

For preparation of *N*-haloaryl­sulfonamides, see: Gowda & Mahadevappa (1983 ▶). For studies of the effect of substituents on the structures of *N*-haloaryl­sulfonamides, see: George *et al.* (2000 ▶); Gowda *et al.* (2007 ▶, 2011*a* ▶,*b* ▶); Olmstead & Power (1986 ▶).
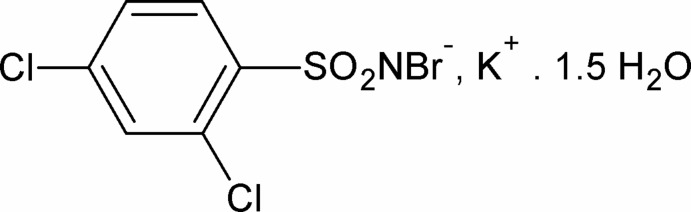


## Experimental

### 

#### Crystal data

K^+^·C_6_H_3_BrCl_2_NO_2_S^−^·1.5H_2_O
*M* *_r_* = 740.18
Monoclinic, 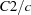

*a* = 12.5263 (7) Å
*b* = 6.7638 (4) Å
*c* = 29.703 (2) Å
β = 98.352 (5)°
*V* = 2489.9 (3) Å^3^
*Z* = 4
Mo *K*α radiation
μ = 4.22 mm^−1^
*T* = 293 K
0.32 × 0.32 × 0.28 mm

#### Data collection

Oxford Diffraction Xcalibur diffractometer with Sapphire CCD detector
Absorption correction: multi-scan (*CrysAlis RED*; Oxford Diffraction, 2009 ▶) *T*_min_ = 0.345, *T*_max_ = 0.384
4960 measured reflections
2535 independent reflections
2204 reflections with *I* > 2σ(*I*)
*R*_int_ = 0.014

#### Refinement

*R*[*F* ^2^ > 2σ(*F* ^2^)] = 0.037
*wR*(*F* ^2^) = 0.093
*S* = 1.09
2535 reflections
150 parameters
3 restraints
H atoms treated by a mixture of independent and constrained refinement
Δρ_max_ = 0.76 e Å^−3^
Δρ_min_ = −0.65 e Å^−3^

### 

Data collection: *CrysAlis CCD* (Oxford Diffraction, 2009 ▶); cell refinement: *CrysAlis CCD*; data reduction: *CrysAlis RED* (Oxford Diffraction, 2009 ▶); program(s) used to solve structure: *SHELXS97* (Sheldrick, 2008 ▶); program(s) used to refine structure: *SHELXL97* (Sheldrick, 2008 ▶); molecular graphics: *PLATON* (Spek, 2009 ▶); software used to prepare material for publication: *SHELXL97*.

## Supplementary Material

Crystal structure: contains datablock(s) I, global. DOI: 10.1107/S1600536812042456/nc2295sup1.cif

Structure factors: contains datablock(s) I. DOI: 10.1107/S1600536812042456/nc2295Isup2.hkl

Additional supplementary materials:  crystallographic information; 3D view; checkCIF report

## Figures and Tables

**Table 1:** Hydrogen-bond geometry (Å, °)

*D*—H⋯*A*	*D*—H	H⋯*A*	*D*⋯*A*	*D*—H⋯*A*
O3—H31⋯Br1^i^	0.80 (2)	2.78 (2)	3.550 (3)	160 (4)
O3—H32⋯N1	0.81 (2)	2.15 (3)	2.917 (4)	158 (5)
O4—H41⋯N1^ii^	0.82 (2)	2.16 (2)	2.957 (3)	165 (5)

Symmetry codes: (i) 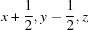; (ii) 
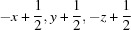
.

**Fig. 1.**
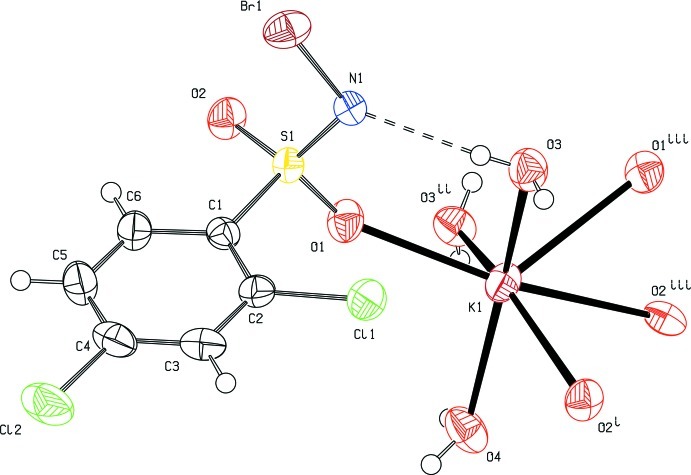
Molecular structure of the title compound, showing the atom labelling scheme for the asymmetric unit and extended to show the coordination geometry for the K+. The displacement ellipsoids are drawn at the 50% probability level. The H atoms are represented as small spheres of arbitrary radii.

**Fig. 2.**
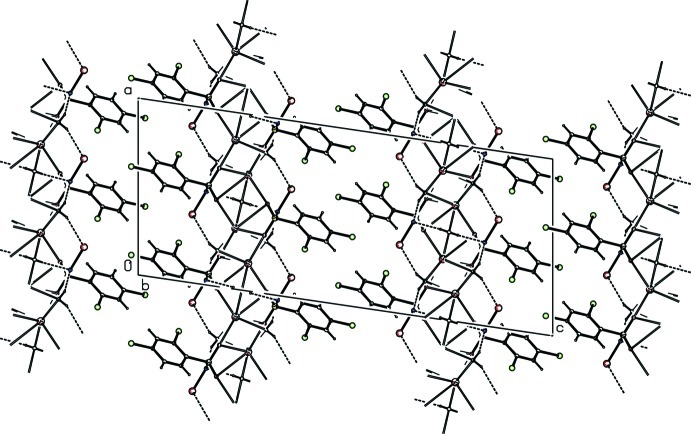
Molecular packing of the title compound with hydrogen bonding shown as dashed lines.

## supplementary crystallographic information

### Comment

The present work was undertaken in order to explore the effect of replacing sodium ion by potassium ion on the solid state structures of metal salts of *N*-haloarylsulfonamidates (Gowda *et al.*, 2007, 2011*a*,*b*). As part of this work, the structure of potassium *N*-bromo-2,4-dichlorobenzenesulfonamidate sesquihydrate (I) has been determined (Fig. 1). The structure of (I) resembles those of potassium *N*-bromo-2-chlorobenzenesulfonamidate sesquihydrate (II) (Gowda *et al.*, 2011*a*), potassium *N*-bromo-4-chlorobenzenesulfonamidate sesquihydrate (III) (Gowda *et al.*, 2011*b*), sodium *N*-bromo-2,4-dichlorobenzenesulfonamidate sesquihydrate (IV) (Gowda *et al.*, 2007) and other sodium *N*-chloro-arylsulfonamidates (George *et al.*, 2000; Olmstead & Power, 1986).

In the title compound, K^+^ ion is hepta coordinated by three O atoms from water molecules and four sulfonyl O atoms of three different *N*-bromo-2,4-dichlorobenzenesulfonamide anions. The replacement of Na^+^ by K^+^ changes co-ordination from hexa to hepta in the structure (Gowda *et al.*, 2007) and other parameters.

The S—N distance of 1.575 (3) Å is consistent with a S—N double bond and is in agreement with the observed values of 1.582 (4) Å in (II), 1.584 (6) Å in (III) and 1.590 (6) Å in (IV).

The asymmetric unit of (I) consists of one potassium cation, one *N*-bromo-2,4-dichlorobenzenesulfonamidate anion and one water molecule in general position and and one water molecule located on a twofold rotation axis.

In the crystal structure the anions are linked by intermolecular O—H···Br and O—H···N hydrogen bonding into layers, that are parallel to the *ac* plane (Fig. 2 and Table 1).

### Experimental

The title compound was prepared by a method similar to the one described by Gowda & Mahadevappa (Gowda & Mahadevappa, 1983). 2 g of 2,4-dichlorobenzenesulfonamide was dissolved with stirring in 40 ml of 5*M* KOH at room temperature. The resultant solution was cooled in ice and 4 ml of liquid bromine was added drop wise with constant stirring. The resultant potassium salt of *N*-bromo-2,4-dichlorobenzenesulfonamide was filtered under suction, washed quickly with a minimum quantity of ice cold water. The purity of the compound was checked by determining its melting point (203–205° C) and estimating, iodometrically, the amount of active bromine present in it. It was further characterized from its infrared spectrum.

Prism like yellow single crystals of the title compound used in the X-ray diffraction studies were obtained from its aqueous solution at room temperature.

### Refinement

H atoms bonded to C were positioned with idealized geometry using a riding model with the aromatic C—H = 0.93 Å. The H atoms bound to O atoms were located in difference map and later restrained to O—H = 0.82 (2) Å. All H atoms were refined with isotropic displacement parameters set at 1.2 *U*_eq_ of the parent atom.

### Crystal data

**Table:**

K^+^·C_6_H_3_BrCl_2_NO_2_S^−^·1.5H_2_O	*F*(000) = 1448
*M**_r_* = 740.18	*D*_x_ = 1.975 Mg m^−^^3^
Monoclinic, *C*2/*c*	Mo *K*α radiation, λ = 0.71073 Å
Hall symbol: -C 2yc	Cell parameters from 2489 reflections
*a* = 12.5263 (7) Å	θ = 3.0–27.7°
*b* = 6.7638 (4) Å	µ = 4.22 mm^−^^1^
*c* = 29.703 (2) Å	*T* = 293 K
β = 98.352 (5)°	Prism, yellow
*V* = 2489.9 (3) Å^3^	0.32 × 0.32 × 0.28 mm
*Z* = 4	

### Data collection

**Table:**

Oxford Diffraction Xcalibur diffractometer with Sapphire CCD detector	2535 independent reflections
Radiation source: fine-focus sealed tube	2204 reflections with *I* > 2σ(*I*)
Graphite monochromator	*R*_int_ = 0.014
Rotation method data acquisition using ω scans.	θ_max_ = 26.4°, θ_min_ = 3.3°
Absorption correction: multi-scan (*CrysAlis RED*; Oxford Diffraction, 2009)	*h* = −15→13
*T*_min_ = 0.345, *T*_max_ = 0.384	*k* = −8→5
4960 measured reflections	*l* = −37→22

### Refinement

**Table:**

Refinement on *F*^2^	Primary atom site location: structure-invariant direct methods
Least-squares matrix: full	Secondary atom site location: difference Fourier map
*R*[*F*^2^ > 2σ(*F*^2^)] = 0.037	Hydrogen site location: inferred from neighbouring sites
*wR*(*F*^2^) = 0.093	H atoms treated by a mixture of independent and constrained refinement
*S* = 1.09	*w* = 1/[σ^2^(*F*_o_^2^) + (0.0429*P*)^2^ + 8.3581*P*] where *P* = (*F*_o_^2^ + 2*F*_c_^2^)/3
2535 reflections	(Δ/σ)_max_ = 0.001
150 parameters	Δρ_max_ = 0.76 e Å^−^^3^
3 restraints	Δρ_min_ = −0.65 e Å^−^^3^

### Special details

**Table:**

Experimental. Empirical absorption correction using spherical harmonics, implemented in SCALE3 ABSPACK scaling algorithm.
Geometry. All e.s.d.'s (except the e.s.d. in the dihedral angle between two l.s. planes) are estimated using the full covariance matrix. The cell e.s.d.'s are taken into account individually in the estimation of e.s.d.'s in distances, angles and torsion angles; correlations between e.s.d.'s in cell parameters are only used when they are defined by crystal symmetry. An approximate (isotropic) treatment of cell e.s.d.'s is used for estimating e.s.d.'s involving l.s. planes.
Refinement. Refinement of *F*^2^ against ALL reflections. The weighted *R*-factor *wR* and goodness of fit *S* are based on *F*^2^, conventional *R*-factors *R* are based on *F*, with *F* set to zero for negative *F*^2^. The threshold expression of *F*^2^ > σ(*F*^2^) is used only for calculating *R*-factors(gt) *etc*. and is not relevant to the choice of reflections for refinement. *R*-factors based on *F*^2^ are statistically about twice as large as those based on *F*, and *R*- factors based on ALL data will be even larger.

### Fractional atomic coordinates and isotropic or equivalent isotropic displacement parameters (Å^2^)

**Table:**

	*x*	*y*	*z*	*U*_iso_*/*U*_eq_	
C1	0.0780 (3)	0.4566 (5)	0.11591 (11)	0.0271 (7)	
C2	0.1438 (3)	0.3775 (5)	0.08637 (12)	0.0317 (7)	
C3	0.1538 (3)	0.4716 (6)	0.04598 (12)	0.0392 (9)	
H3	0.1980	0.4192	0.0264	0.047*	
C4	0.0974 (3)	0.6446 (6)	0.03496 (13)	0.0426 (9)	
C5	0.0320 (3)	0.7248 (6)	0.06337 (14)	0.0447 (9)	
H5	−0.0056	0.8413	0.0556	0.054*	
C6	0.0228 (3)	0.6302 (5)	0.10361 (13)	0.0367 (8)	
H6	−0.0214	0.6841	0.1230	0.044*	
Br1	−0.11245 (3)	0.10625 (6)	0.130655 (14)	0.04440 (14)	
Cl1	0.21550 (9)	0.15999 (15)	0.09788 (4)	0.0485 (3)	
Cl2	0.10726 (10)	0.7571 (2)	−0.01671 (4)	0.0650 (4)	
K1	0.34306 (6)	0.13766 (12)	0.23492 (3)	0.0366 (2)	
N1	0.0277 (2)	0.1274 (4)	0.16389 (10)	0.0319 (6)	
O1	0.1712 (2)	0.3416 (4)	0.19584 (8)	0.0386 (6)	
O2	−0.0093 (2)	0.4804 (4)	0.18845 (8)	0.0383 (6)	
O3	0.2037 (2)	−0.1486 (4)	0.19206 (10)	0.0426 (6)	
H31	0.234 (3)	−0.195 (7)	0.1725 (12)	0.051*	
H32	0.155 (3)	−0.088 (6)	0.1774 (14)	0.051*	
O4	0.5000	0.4277 (6)	0.2500	0.0471 (10)	
H41	0.490 (4)	0.502 (6)	0.2710 (11)	0.056*	
S1	0.06429 (7)	0.34987 (12)	0.16983 (3)	0.02745 (19)	

### Atomic displacement parameters (Å^2^)

**Table:**

	*U*^11^	*U*^22^	*U*^33^	*U*^12^	*U*^13^	*U*^23^
C1	0.0271 (16)	0.0256 (16)	0.0280 (16)	−0.0039 (13)	0.0014 (12)	−0.0003 (13)
C2	0.0261 (16)	0.0319 (18)	0.0371 (18)	−0.0031 (14)	0.0042 (14)	0.0000 (15)
C3	0.0350 (19)	0.048 (2)	0.0364 (19)	−0.0108 (17)	0.0109 (15)	−0.0008 (17)
C4	0.040 (2)	0.048 (2)	0.038 (2)	−0.0161 (18)	−0.0006 (16)	0.0113 (17)
C5	0.047 (2)	0.036 (2)	0.050 (2)	0.0043 (18)	0.0016 (18)	0.0116 (18)
C6	0.0359 (19)	0.0322 (18)	0.041 (2)	0.0040 (15)	0.0036 (15)	0.0029 (15)
Br1	0.0342 (2)	0.0494 (3)	0.0498 (2)	−0.00725 (17)	0.00672 (16)	−0.01193 (18)
Cl1	0.0484 (6)	0.0376 (5)	0.0645 (7)	0.0110 (4)	0.0250 (5)	0.0029 (5)
Cl2	0.0592 (7)	0.0857 (9)	0.0483 (6)	−0.0170 (6)	0.0024 (5)	0.0322 (6)
K1	0.0312 (4)	0.0354 (4)	0.0423 (4)	0.0051 (3)	0.0021 (3)	−0.0034 (3)
N1	0.0307 (15)	0.0279 (15)	0.0373 (16)	0.0006 (12)	0.0060 (12)	0.0047 (12)
O1	0.0373 (14)	0.0399 (14)	0.0353 (14)	0.0026 (11)	−0.0064 (11)	0.0010 (11)
O2	0.0444 (15)	0.0387 (14)	0.0339 (13)	0.0094 (12)	0.0130 (11)	−0.0046 (11)
O3	0.0475 (17)	0.0380 (15)	0.0434 (16)	0.0052 (12)	0.0099 (13)	0.0004 (12)
O4	0.073 (3)	0.031 (2)	0.041 (2)	0.000	0.022 (2)	0.000
S1	0.0302 (4)	0.0260 (4)	0.0260 (4)	0.0028 (3)	0.0033 (3)	0.0000 (3)

### Geometric parameters (Å, º)

**Table:**

C1—C6	1.384 (5)	K1—O3	2.788 (3)
C1—C2	1.395 (5)	K1—O1^iii^	2.895 (3)
C1—S1	1.787 (3)	K1—O2^iii^	3.045 (3)
C2—C3	1.380 (5)	K1—S1^iii^	3.4910 (12)
C2—Cl1	1.732 (4)	N1—S1	1.575 (3)
C3—C4	1.381 (6)	O1—S1	1.447 (3)
C3—H3	0.9300	O1—K1^ii^	2.895 (3)
C4—C5	1.370 (6)	O2—S1	1.443 (3)
C4—Cl2	1.734 (4)	O2—K1^iv^	2.683 (2)
C5—C6	1.375 (5)	O2—K1^ii^	3.045 (3)
C5—H5	0.9300	O3—K1^iii^	2.740 (3)
C6—H6	0.9300	O3—H31	0.802 (19)
Br1—N1	1.890 (3)	O3—H32	0.808 (19)
K1—O1	2.675 (3)	O4—K1^v^	2.767 (3)
K1—O2^i^	2.683 (2)	O4—H41	0.820 (19)
K1—O3^ii^	2.740 (3)	S1—K1^ii^	3.4910 (12)
K1—O4	2.767 (3)		
			
C6—C1—C2	118.5 (3)	O3—K1—K1^v^	130.38 (6)
C6—C1—S1	118.0 (3)	O1^iii^—K1—K1^v^	90.06 (6)
C2—C1—S1	123.4 (3)	O2^iii^—K1—K1^v^	43.27 (5)
C3—C2—C1	120.4 (3)	S1^iii^—K1—K1^v^	66.97 (2)
C3—C2—Cl1	117.0 (3)	O1—K1—K1^iii^	94.01 (7)
C1—C2—Cl1	122.6 (3)	O2^i^—K1—K1^iii^	103.41 (6)
C2—C3—C4	119.3 (4)	O3^ii^—K1—K1^iii^	93.78 (7)
C2—C3—H3	120.4	O4—K1—K1^iii^	157.36 (5)
C4—C3—H3	120.4	O3—K1—K1^iii^	38.92 (6)
C5—C4—C3	121.4 (4)	O1^iii^—K1—K1^iii^	38.01 (5)
C5—C4—Cl2	119.8 (3)	O2^iii^—K1—K1^iii^	84.35 (5)
C3—C4—Cl2	118.8 (3)	S1^iii^—K1—K1^iii^	61.08 (2)
C4—C5—C6	118.9 (4)	K1^v^—K1—K1^iii^	120.90 (2)
C4—C5—H5	120.5	O1—K1—K1^ii^	41.78 (6)
C6—C5—H5	120.5	O2^i^—K1—K1^ii^	149.20 (6)
C5—C6—C1	121.5 (4)	O3^ii^—K1—K1^ii^	39.74 (6)
C5—C6—H6	119.2	O4—K1—K1^ii^	78.55 (6)
C1—C6—H6	119.2	O3—K1—K1^ii^	108.70 (7)
O1—K1—O2^i^	123.54 (8)	O1^iii^—K1—K1^ii^	107.85 (6)
O1—K1—O3^ii^	79.66 (9)	O2^iii^—K1—K1^ii^	117.01 (6)
O2^i^—K1—O3^ii^	149.20 (9)	S1^iii^—K1—K1^ii^	113.48 (3)
O1—K1—O4	102.28 (8)	K1^v^—K1—K1^ii^	120.90 (2)
O2^i^—K1—O4	80.66 (7)	K1^iii^—K1—K1^ii^	104.55 (3)
O3^ii^—K1—O4	74.11 (7)	S1—N1—Br1	111.33 (16)
O1—K1—O3	75.45 (8)	S1—O1—K1	151.14 (16)
O2^i^—K1—O3	85.56 (9)	S1—O1—K1^ii^	101.80 (13)
O3^ii^—K1—O3	122.44 (5)	K1—O1—K1^ii^	100.21 (8)
O4—K1—O3	161.68 (7)	S1—O2—K1^iv^	165.01 (16)
O1—K1—O1^iii^	122.42 (5)	S1—O2—K1^ii^	95.45 (12)
O2^i^—K1—O1^iii^	102.09 (8)	K1^iv^—O2—K1^ii^	85.68 (7)
O3^ii^—K1—O1^iii^	76.13 (8)	K1^iii^—O3—K1	101.34 (10)
O4—K1—O1^iii^	119.42 (6)	K1^iii^—O3—H31	123 (4)
O3—K1—O1^iii^	75.21 (8)	K1—O3—H31	106 (4)
O1—K1—O2^iii^	157.55 (8)	K1^iii^—O3—H32	117 (4)
O2^i^—K1—O2^iii^	78.37 (9)	K1—O3—H32	106 (3)
O3^ii^—K1—O2^iii^	78.12 (8)	H31—O3—H32	102 (5)
O4—K1—O2^iii^	74.58 (7)	K1—O4—K1^v^	89.71 (12)
O3—K1—O2^iii^	114.43 (8)	K1—O4—H41	112 (3)
O1^iii^—K1—O2^iii^	48.19 (7)	K1^v^—O4—H41	119 (3)
O1—K1—S1^iii^	142.27 (7)	O2—S1—O1	114.34 (16)
O2^i^—K1—S1^iii^	91.31 (6)	O2—S1—N1	115.83 (16)
O3^ii^—K1—S1^iii^	74.74 (7)	O1—S1—N1	104.73 (16)
O4—K1—S1^iii^	96.86 (5)	O2—S1—C1	104.25 (15)
O3—K1—S1^iii^	95.47 (7)	O1—S1—C1	107.00 (16)
O1^iii^—K1—S1^iii^	23.94 (5)	N1—S1—C1	110.46 (16)
O2^iii^—K1—S1^iii^	24.30 (5)	O2—S1—K1^ii^	60.25 (11)
O1—K1—K1^v^	145.05 (6)	O1—S1—K1^ii^	54.26 (11)
O2^i^—K1—K1^v^	51.05 (6)	N1—S1—K1^ii^	132.87 (12)
O3^ii^—K1—K1^v^	98.18 (7)	C1—S1—K1^ii^	115.93 (11)
O4—K1—K1^v^	45.15 (6)		
			
C6—C1—C2—C3	0.3 (5)	S1^iii^—K1—O3—K1^iii^	26.89 (8)
S1—C1—C2—C3	−177.6 (3)	K1^v^—K1—O3—K1^iii^	91.14 (10)
C6—C1—C2—Cl1	−179.3 (3)	K1^ii^—K1—O3—K1^iii^	−90.17 (8)
S1—C1—C2—Cl1	2.9 (4)	O1—K1—O4—K1^v^	−165.19 (7)
C1—C2—C3—C4	−0.3 (5)	O2^i^—K1—O4—K1^v^	−42.70 (6)
Cl1—C2—C3—C4	179.3 (3)	O3^ii^—K1—O4—K1^v^	119.46 (7)
C2—C3—C4—C5	0.1 (6)	O3—K1—O4—K1^v^	−84.4 (3)
C2—C3—C4—Cl2	−178.0 (3)	O1^iii^—K1—O4—K1^v^	55.99 (7)
C3—C4—C5—C6	0.0 (6)	O2^iii^—K1—O4—K1^v^	37.71 (5)
Cl2—C4—C5—C6	178.1 (3)	S1^iii^—K1—O4—K1^v^	47.51 (2)
C4—C5—C6—C1	−0.1 (6)	K1^iii^—K1—O4—K1^v^	59.77 (14)
C2—C1—C6—C5	−0.1 (5)	K1^ii^—K1—O4—K1^v^	160.16 (4)
S1—C1—C6—C5	177.9 (3)	K1^iv^—O2—S1—O1	−89.2 (6)
O2^i^—K1—O1—S1	77.2 (4)	K1^ii^—O2—S1—O1	4.50 (17)
O3^ii^—K1—O1—S1	−124.9 (3)	K1^iv^—O2—S1—N1	32.8 (7)
O4—K1—O1—S1	164.0 (3)	K1^ii^—O2—S1—N1	126.44 (14)
O3—K1—O1—S1	2.7 (3)	K1^iv^—O2—S1—C1	154.3 (6)
O1^iii^—K1—O1—S1	−58.8 (3)	K1^ii^—O2—S1—C1	−111.99 (12)
O2^iii^—K1—O1—S1	−116.7 (3)	K1^iv^—O2—S1—K1^ii^	−93.7 (6)
S1^iii^—K1—O1—S1	−77.2 (4)	K1—O1—S1—O2	134.2 (3)
K1^v^—K1—O1—S1	145.6 (3)	K1^ii^—O1—S1—O2	−4.81 (18)
K1^iii^—K1—O1—S1	−31.8 (3)	K1—O1—S1—N1	6.3 (4)
K1^ii^—K1—O1—S1	−139.2 (4)	K1^ii^—O1—S1—N1	−132.65 (13)
O2^i^—K1—O1—K1^ii^	−143.58 (9)	K1—O1—S1—C1	−111.0 (3)
O3^ii^—K1—O1—K1^ii^	14.32 (8)	K1^ii^—O1—S1—C1	110.07 (14)
O4—K1—O1—K1^ii^	−56.74 (8)	K1—O1—S1—K1^ii^	139.0 (4)
O3—K1—O1—K1^ii^	141.96 (10)	Br1—N1—S1—O2	52.7 (2)
O1^iii^—K1—O1—K1^ii^	80.46 (13)	Br1—N1—S1—O1	179.61 (16)
O2^iii^—K1—O1—K1^ii^	22.6 (2)	Br1—N1—S1—C1	−65.5 (2)
S1^iii^—K1—O1—K1^ii^	62.01 (13)	Br1—N1—S1—K1^ii^	125.06 (12)
K1^v^—K1—O1—K1^ii^	−75.18 (13)	C6—C1—S1—O2	1.3 (3)
K1^iii^—K1—O1—K1^ii^	107.44 (7)	C2—C1—S1—O2	179.2 (3)
O1—K1—O3—K1^iii^	−115.86 (10)	C6—C1—S1—O1	−120.2 (3)
O2^i^—K1—O3—K1^iii^	117.79 (9)	C2—C1—S1—O1	57.7 (3)
O3^ii^—K1—O3—K1^iii^	−48.49 (13)	C6—C1—S1—N1	126.4 (3)
O4—K1—O3—K1^iii^	159.0 (2)	C2—C1—S1—N1	−55.8 (3)
O1^iii^—K1—O3—K1^iii^	14.03 (8)	C6—C1—S1—K1^ii^	−62.2 (3)
O2^iii^—K1—O3—K1^iii^	42.71 (11)	C2—C1—S1—K1^ii^	115.6 (3)

Symmetry codes: (i) *x*+1/2, *y*−1/2, *z*; (ii) −*x*+1/2, *y*+1/2, −*z*+1/2; (iii) −*x*+1/2, *y*−1/2, −*z*+1/2; (iv) *x*−1/2, *y*+1/2, *z*; (v) −*x*+1, *y*, −*z*+1/2.

### Hydrogen-bond geometry (Å, º)

**Table:**

*D*—H···*A*	*D*—H	H···*A*	*D*···*A*	*D*—H···*A*
O3—H31···Br1^i^	0.80 (2)	2.78 (2)	3.550 (3)	160 (4)
O3—H32···N1	0.81 (2)	2.15 (3)	2.917 (4)	158 (5)
O4—H41···N1^ii^	0.82 (2)	2.16 (2)	2.957 (3)	165 (5)

Symmetry codes: (i) *x*+1/2, *y*−1/2, *z*; (ii) −*x*+1/2, *y*+1/2, −*z*+1/2.
